# 1-(2-Cyano­ethyl)-2-(2-pyrid­yl)-1*H*,3*H*-benzimidazol-3-ium perchlorate

**DOI:** 10.1107/S1600536809055251

**Published:** 2010-01-20

**Authors:** Yan Li, Xiaoliang Tang, Jiayu Chen, Daxiang Wu, Weisheng Liu

**Affiliations:** a Key Laboratory of Nonferrous Metal Chemistry and Resources Utilization of Gansu Province, College of Chemistry and Chemical Engineering and State Key Laboratory of Applied Organic Chemistry, Lanzhou University, Lanzhou 730000, People’s Republic of China; b Teaching and Research Department, Urumqi Command College, The Chinese People’s Armed Police Forces, Urumqi 830049, People’s Republic of China

## Abstract

The title compound, C_15_H_13_N_4_^+^·ClO_4_^−^, comprises a nonplanar 1-(2-cyano­ethyl)-2-(2-pyrid­yl)-1*H*,3*H*-benzimidazol-3-ium cation [dihedral angle between the imidazole and pyridine rings = 22.5 (8)°] and a perchlorate anion. The cation is formed by protonation of the N atom of the benzimidazole ring. A charged N—H⋯O hydrogen bond connects the anion and cation, and inter­molecular C—H⋯O and C—H⋯N inter­actions contribute to the crystal packing.

## Related literature

For the pharmacological activity of benzimidazole and its derivatives, see: Feng & Xu (2001 ▶); Ferey (2001 ▶); Hossain *et al.* (2001 ▶); Howarth & Hanlon (2001 ▶); Kazak *et al.* (2006 ▶); Li *et al.* (1998 ▶).
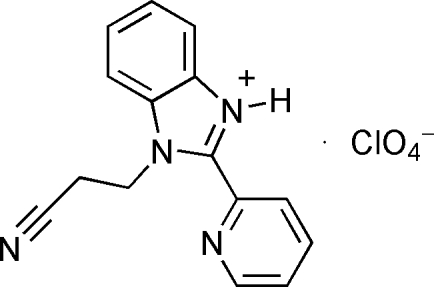


## Experimental

### 

#### Crystal data

C_15_H_13_N_4_^+^·ClO_4_^−^
*M* *_r_* = 348.74
Triclinic, 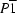

*a* = 8.788 (1) Å
*b* = 9.4608 (10) Å
*c* = 10.6013 (11) Å
α = 69.690 (2)°
β = 73.844 (2)°
γ = 86.193 (2)°
*V* = 793.52 (15) Å^3^
*Z* = 2
Mo *K*α radiation
μ = 0.27 mm^−1^
*T* = 296 K
0.22 × 0.21 × 0.19 mm

#### Data collection

Bruker SMART CCD area-detector diffractometer
Absorption correction: multi-scan (*SADABS*; Bruker, 1997 ▶) *T*_min_ = 0.940, *T*_max_ = 0.955
4167 measured reflections
2924 independent reflections
2316 reflections with *I* > 2σ(*I*)
*R*_int_ = 0.013

#### Refinement

*R*[*F* ^2^ > 2σ(*F* ^2^)] = 0.047
*wR*(*F* ^2^) = 0.122
*S* = 1.01
2924 reflections
221 parameters
102 restraints
H atoms treated by a mixture of independent and constrained refinement
Δρ_max_ = 0.35 e Å^−3^
Δρ_min_ = −0.24 e Å^−3^

### 

Data collection: *SMART* (Bruker, 1997 ▶); cell refinement: *SAINT* (Bruker, 1997 ▶); data reduction: *SAINT*; program(s) used to solve structure: *SHELXS97* (Sheldrick, 2008 ▶); program(s) used to refine structure: *SHELXL97* (Sheldrick, 2008 ▶); molecular graphics: *SHELXTL* (Sheldrick, 2008 ▶); software used to prepare material for publication: *SHELXTL*.

## Supplementary Material

Crystal structure: contains datablocks I, global. DOI: 10.1107/S1600536809055251/kp2242sup1.cif

Structure factors: contains datablocks I. DOI: 10.1107/S1600536809055251/kp2242Isup2.hkl

Additional supplementary materials:  crystallographic information; 3D view; checkCIF report

## Figures and Tables

**Table 1:** Hydrogen-bond geometry (Å, °)

*D*—H⋯*A*	*D*—H	H⋯*A*	*D*⋯*A*	*D*—H⋯*A*
N2—H5⋯O4	0.77 (3)	2.11 (3)	2.885 (4)	179 (4)
C11—H11⋯O3	0.93	2.54	3.343 (5)	145
C4—H4⋯O4	0.93	2.62	3.347 (4)	135
C13—H13*A*⋯N1	0.97	2.41	2.903 (4)	111
C10—H10⋯N4^i^	0.93	2.64	3.423 (4)	142
C13—H13*B*⋯O2^ii^	0.97	2.60	3.427 (4)	144
C14—H14*B*⋯O2^iii^	0.97	2.57	3.483 (4)	157
C2—H2⋯O1^iv^	0.93	2.62	3.552 (4)	180

Symmetry codes: (i) 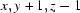; (ii) 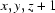; (iii) 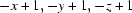; (iv) 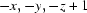.

**Fig. 1.**
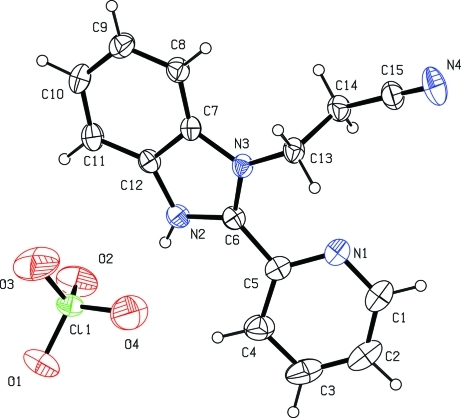
Molecular structure of the title compound. Displacement ellipsoids are drawn at the 30% probability level.

**Fig. 2.**
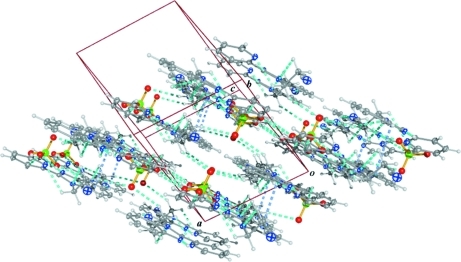
The three-dimensional packing diagram of the compound. Hydrogen bonds are shown as turquiose dashed lines. H atoms not involved in hydrogen bonding have been omitted for clarity. The π···π interaction between benzimidazole groups are shown as light blue dashed lines.

## supplementary crystallographic information

### Comment

For many years benzimidazole and its derivatives continue to attract attention in chemical synthesis, structural science and applied biological research due to their various pharmacological activities (Li *et al.*, 1998; Howarth & Hanlon, 2001; Feng & Xu, 2001; Ferey, 2001; Kazak *et al.*, 2006). Like the phenanthroline based ligands these compounds are known to chelate metal atoms although their coordination chemistry has not been as extensively explored. To further widen the scope of research on the chemical and physical properties of benzimidazole derivatives, there is a need to prepare a new series of benzimidazole derivatives. In this paper, we present the structure of the title compound as a perchlorate salt.

The compound is composed of C_15_H_13_N_4_^+^ cation and a perchlorate anion, in a 1:1 ratio (Fig. 1). In C_15_H_13_N_4_^+^ cation, the molecular skeleton of protonated 2-(2-pyridyl)benzimidazole is non-planar and the dihedral angle between benzimidazole ring and pyridine ring is 25.46°. Two of O atoms of the perchlorate anion, link benzimidazole *via* the charged N(2)–H(5)···O(4) and C(11)–H(11)···O(3) hydrogen bonds (Table 1). In addition, intermolecular C–H···O, N–H···O hydrogen bonds and π···π interactions between benzimidazole groups [*Cg*···*Cg*^i^ plane···plane separation = 3.6933 (16) Å, i = 1 - *x*, 1 - *y*, 1 - *z*] link the molecules into a three-dimensional structure (Fig. 2).

### Experimental

The ligand, 1-(cyanoethyl)-2-(2-pyridyl)benzimidazole was prepared according to the method described by Hossain *et al.* (2001), using 2-(2-pyridyl)benzimidazole and acrylonitrile as starting materials (yield: 62%, m.p. 378–383 K).

Single crystals of the title compound suitable for X-ray analysis were obtained by slow evaporation of a methanol solution (6 ml) containing Fe(ClO_4_)_3_.10H_2_O (26.7 mg, 0.05 mmol) and 1-(cyanoethyl)-2-(2-pyridyl)benzimidazole (24.8 mg, 0.10 mmol) after two weeks at room temperature. Our attempt to obtain an iron(III) perchlorate complex of the ligand failed and, instead, the protonated ligand salt was crystallised.

### Refinement

The position of the amine H atom was refined freely, together with its individual isotropic displacement parameter. All other H atoms were positioned geometrically with C—H = 0.93 and 0.97 Å for aromatic, methylene H atoms, respectively, and constrained to ride on their parent atoms with *U*iso(H) = 1.2*U*eq(C).

### Crystal data

**Table:**

C_15_H_13_N_4_^+^·ClO_4_^−^	*Z* = 2
*M**_r_* = 348.74	*F*(000) = 360
Triclinic, *P*1	*D*_x_ = 1.460 Mg m^−^^3^
Hall symbol: -P 1	Mo *K*α radiation, λ = 0.71073 Å
*a* = 8.788 (1) Å	Cell parameters from 1758 reflections
*b* = 9.4608 (10) Å	θ = 2.4–26.4°
*c* = 10.6013 (11) Å	µ = 0.27 mm^−^^1^
α = 69.690 (2)°	*T* = 296 K
β = 73.844 (2)°	Block, yellow
γ = 86.193 (2)°	0.22 × 0.21 × 0.19 mm
*V* = 793.52 (15) Å^3^	

### Data collection

**Table:**

Bruker SMART CCD area-detector diffractometer	2924 independent reflections
Radiation source: fine-focus sealed tube	2316 reflections with *I* > 2σ(*I*)
graphite	*R*_int_ = 0.013
phi and ω scans	θ_max_ = 25.5°, θ_min_ = 2.1°
Absorption correction: multi-scan (*SADABS*; Bruker, 1997)	*h* = −10→10
*T*_min_ = 0.940, *T*_max_ = 0.955	*k* = −11→6
4167 measured reflections	*l* = −12→12

### Refinement

**Table:**

Refinement on *F*^2^	Primary atom site location: structure-invariant direct methods
Least-squares matrix: full	Secondary atom site location: difference Fourier map
*R*[*F*^2^ > 2σ(*F*^2^)] = 0.047	Hydrogen site location: inferred from neighbouring sites
*wR*(*F*^2^) = 0.122	H atoms treated by a mixture of independent and constrained refinement
*S* = 1.01	*w* = 1/[σ^2^(*F*_o_^2^) + (0.043*P*)^2^ + 0.670*P*] where *P* = (*F*_o_^2^ + 2*F*_c_^2^)/3
2924 reflections	(Δ/σ)_max_ < 0.001
221 parameters	Δρ_max_ = 0.35 e Å^−^^3^
102 restraints	Δρ_min_ = −0.24 e Å^−^^3^

### Special details

**Table:**

Geometry. All e.s.d.'s (except the e.s.d. in the dihedral angle between two l.s. planes) are estimated using the full covariance matrix. The cell e.s.d.'s are taken into account individually in the estimation of e.s.d.'s in distances, angles and torsion angles; correlations between e.s.d.'s in cell parameters are only used when they are defined by crystal symmetry. An approximate (isotropic) treatment of cell e.s.d.'s is used for estimating e.s.d.'s involving l.s. planes.
Refinement. Refinement of *F*^2^ against ALL reflections. The weighted *R*-factor *wR* and goodness of fit *S* are based on *F*^2^, conventional *R*-factors *R* are based on *F*, with *F* set to zero for negative *F*^2^. The threshold expression of *F*^2^ > σ(*F*^2^) is used only for calculating *R*-factors(gt) *etc*. and is not relevant to the choice of reflections for refinement. *R*-factors based on *F*^2^ are statistically about twice as large as those based on *F*, and *R*- factors based on ALL data will be even larger.

### Fractional atomic coordinates and isotropic or equivalent isotropic displacement parameters (Å^2^)

**Table:**

	*x*	*y*	*z*	*U*_iso_*/*U*_eq_	
C1	0.1586 (4)	−0.0845 (4)	0.8393 (4)	0.0755 (9)	
H1	0.1941	−0.1600	0.9072	0.091*	
C2	0.0604 (4)	−0.1248 (4)	0.7774 (4)	0.0781 (10)	
H2	0.0314	−0.2256	0.8016	0.094*	
C3	0.0053 (4)	−0.0148 (4)	0.6792 (4)	0.0797 (10)	
H3	−0.0635	−0.0396	0.6369	0.096*	
C4	0.0524 (4)	0.1333 (4)	0.6431 (3)	0.0657 (8)	
H4	0.0168	0.2102	0.5762	0.079*	
C5	0.1544 (3)	0.1638 (3)	0.7097 (3)	0.0469 (6)	
C6	0.2150 (3)	0.3172 (3)	0.6751 (2)	0.0409 (6)	
C7	0.3245 (3)	0.5180 (3)	0.6825 (2)	0.0402 (5)	
C8	0.3956 (3)	0.6193 (3)	0.7178 (3)	0.0493 (6)	
H8	0.4122	0.5949	0.8057	0.059*	
C9	0.4403 (4)	0.7580 (3)	0.6163 (3)	0.0578 (7)	
H9	0.4903	0.8288	0.6354	0.069*	
C10	0.4127 (4)	0.7952 (3)	0.4854 (3)	0.0582 (7)	
H10	0.4437	0.8908	0.4199	0.070*	
C11	0.3412 (3)	0.6949 (3)	0.4506 (3)	0.0516 (7)	
H11	0.3223	0.7203	0.3634	0.062*	
C12	0.2988 (3)	0.5544 (3)	0.5516 (2)	0.0410 (6)	
C13	0.2574 (3)	0.2959 (3)	0.9067 (2)	0.0437 (6)	
H13A	0.1756	0.2165	0.9481	0.052*	
H13B	0.2286	0.3688	0.9542	0.052*	
C14	0.4152 (3)	0.2296 (3)	0.9245 (3)	0.0522 (7)	
H14A	0.4470	0.1621	0.8715	0.063*	
H14B	0.4953	0.3102	0.8880	0.063*	
C15	0.4052 (4)	0.1470 (3)	1.0716 (3)	0.0591 (7)	
Cl1	0.17292 (8)	0.51237 (8)	0.19213 (6)	0.0485 (2)	
H5	0.207 (3)	0.416 (3)	0.491 (3)	0.044 (8)*	
N1	0.2065 (3)	0.0574 (3)	0.8078 (3)	0.0601 (6)	
N2	0.2311 (3)	0.4263 (2)	0.5520 (2)	0.0449 (5)	
N3	0.2685 (2)	0.3697 (2)	0.75719 (19)	0.0399 (5)	
N4	0.3982 (4)	0.0807 (4)	1.1839 (3)	0.0887 (10)	
O1	0.0518 (3)	0.5091 (3)	0.1302 (3)	0.0896 (8)	
O2	0.3196 (3)	0.4965 (3)	0.1030 (3)	0.0923 (8)	
O3	0.1764 (4)	0.6462 (4)	0.2192 (4)	0.1268 (12)	
O4	0.1453 (4)	0.3906 (4)	0.3209 (3)	0.1253 (12)	

### Atomic displacement parameters (Å^2^)

**Table:**

	*U*^11^	*U*^22^	*U*^33^	*U*^12^	*U*^13^	*U*^23^
C1	0.087 (2)	0.0490 (17)	0.080 (2)	−0.0088 (16)	−0.0136 (18)	−0.0141 (16)
C2	0.085 (2)	0.0587 (19)	0.081 (2)	−0.0247 (17)	0.0083 (18)	−0.0317 (17)
C3	0.084 (2)	0.088 (2)	0.075 (2)	−0.0308 (19)	−0.0067 (18)	−0.0423 (19)
C4	0.0707 (19)	0.073 (2)	0.0575 (18)	−0.0127 (16)	−0.0179 (15)	−0.0236 (15)
C5	0.0466 (14)	0.0479 (14)	0.0454 (14)	−0.0034 (11)	−0.0045 (11)	−0.0204 (12)
C6	0.0433 (14)	0.0456 (14)	0.0349 (13)	0.0033 (11)	−0.0098 (10)	−0.0159 (11)
C7	0.0443 (13)	0.0380 (13)	0.0352 (13)	0.0049 (10)	−0.0098 (10)	−0.0102 (10)
C8	0.0652 (17)	0.0459 (15)	0.0422 (14)	0.0035 (12)	−0.0224 (13)	−0.0159 (12)
C9	0.0699 (19)	0.0453 (16)	0.0621 (18)	−0.0032 (13)	−0.0218 (15)	−0.0192 (14)
C10	0.0704 (19)	0.0397 (15)	0.0507 (16)	−0.0021 (13)	−0.0122 (14)	−0.0012 (12)
C11	0.0604 (17)	0.0511 (16)	0.0360 (13)	0.0034 (13)	−0.0134 (12)	−0.0061 (12)
C12	0.0418 (13)	0.0454 (14)	0.0354 (13)	0.0038 (11)	−0.0104 (10)	−0.0139 (11)
C13	0.0563 (15)	0.0430 (14)	0.0286 (12)	0.0020 (11)	−0.0118 (11)	−0.0084 (10)
C14	0.0564 (16)	0.0528 (16)	0.0418 (14)	−0.0002 (13)	−0.0165 (12)	−0.0065 (12)
C15	0.0691 (19)	0.0531 (17)	0.0565 (18)	0.0111 (14)	−0.0295 (15)	−0.0124 (14)
Cl1	0.0493 (4)	0.0606 (4)	0.0450 (4)	−0.0011 (3)	−0.0169 (3)	−0.0257 (3)
N1	0.0657 (15)	0.0470 (13)	0.0656 (15)	−0.0028 (11)	−0.0196 (12)	−0.0149 (12)
N2	0.0534 (13)	0.0508 (13)	0.0340 (11)	0.0001 (10)	−0.0164 (10)	−0.0152 (10)
N3	0.0491 (12)	0.0382 (11)	0.0309 (10)	0.0014 (9)	−0.0115 (9)	−0.0096 (9)
N4	0.108 (2)	0.093 (2)	0.0539 (17)	0.0245 (18)	−0.0388 (16)	−0.0032 (16)
O1	0.0684 (15)	0.122 (2)	0.1015 (18)	0.0124 (14)	−0.0503 (14)	−0.0474 (16)
O2	0.0586 (14)	0.138 (2)	0.0961 (18)	0.0062 (14)	−0.0112 (13)	−0.0667 (17)
O3	0.139 (3)	0.116 (2)	0.172 (3)	0.0001 (19)	−0.041 (2)	−0.108 (2)
O4	0.173 (3)	0.129 (2)	0.0615 (16)	−0.058 (2)	−0.0465 (18)	0.0051 (16)

### Geometric parameters (Å, °)

**Table:**

C1—N1	1.333 (4)	C9—H9	0.9300
C1—C2	1.361 (5)	C10—C11	1.373 (4)
C1—H1	0.9300	C10—H10	0.9300
C2—C3	1.365 (5)	C11—C12	1.382 (3)
C2—H2	0.9300	C11—H11	0.9300
C3—C4	1.377 (4)	C12—N2	1.382 (3)
C3—H3	0.9300	C13—N3	1.471 (3)
C4—C5	1.384 (4)	C13—C14	1.517 (4)
C4—H4	0.9300	C13—H13A	0.9700
C5—N1	1.335 (3)	C13—H13B	0.9700
C5—C6	1.465 (3)	C14—C15	1.462 (4)
C6—N2	1.330 (3)	C14—H14A	0.9700
C6—N3	1.336 (3)	C14—H14B	0.9700
C7—C8	1.381 (3)	C15—N4	1.123 (4)
C7—C12	1.389 (3)	Cl1—O3	1.396 (3)
C7—N3	1.393 (3)	Cl1—O1	1.404 (2)
C8—C9	1.374 (4)	Cl1—O2	1.404 (2)
C8—H8	0.9300	Cl1—O4	1.418 (3)
C9—C10	1.395 (4)	N2—H5	0.78 (3)
			
N1—C1—C2	123.6 (3)	C10—C11—H11	121.7
N1—C1—H1	118.2	C12—C11—H11	121.7
C2—C1—H1	118.2	C11—C12—N2	132.5 (2)
C1—C2—C3	118.8 (3)	C11—C12—C7	121.5 (2)
C1—C2—H2	120.6	N2—C12—C7	106.0 (2)
C3—C2—H2	120.6	N3—C13—C14	109.9 (2)
C2—C3—C4	119.5 (3)	N3—C13—H13A	109.7
C2—C3—H3	120.2	C14—C13—H13A	109.7
C4—C3—H3	120.2	N3—C13—H13B	109.7
C3—C4—C5	117.8 (3)	C14—C13—H13B	109.7
C3—C4—H4	121.1	H13A—C13—H13B	108.2
C5—C4—H4	121.1	C15—C14—C13	111.2 (2)
N1—C5—C4	123.1 (3)	C15—C14—H14A	109.4
N1—C5—C6	115.2 (2)	C13—C14—H14A	109.4
C4—C5—C6	121.7 (3)	C15—C14—H14B	109.4
N2—C6—N3	108.6 (2)	C13—C14—H14B	109.4
N2—C6—C5	124.6 (2)	H14A—C14—H14B	108.0
N3—C6—C5	126.7 (2)	N4—C15—C14	178.5 (3)
C8—C7—C12	121.9 (2)	O3—Cl1—O1	111.91 (19)
C8—C7—N3	131.6 (2)	O3—Cl1—O2	109.33 (18)
C12—C7—N3	106.5 (2)	O1—Cl1—O2	109.27 (15)
C9—C8—C7	116.5 (2)	O3—Cl1—O4	108.3 (2)
C9—C8—H8	121.8	O1—Cl1—O4	108.51 (17)
C7—C8—H8	121.8	O2—Cl1—O4	109.5 (2)
C8—C9—C10	121.7 (3)	C1—N1—C5	117.2 (3)
C8—C9—H9	119.2	C6—N2—C12	110.0 (2)
C10—C9—H9	119.2	C6—N2—H5	123 (2)
C11—C10—C9	121.9 (2)	C12—N2—H5	127 (2)
C11—C10—H10	119.1	C6—N3—C7	108.90 (19)
C9—C10—H10	119.1	C6—N3—C13	127.9 (2)
C10—C11—C12	116.6 (2)	C7—N3—C13	122.87 (19)
			
N1—C1—C2—C3	−1.0 (5)	N3—C13—C14—C15	−176.3 (2)
C1—C2—C3—C4	1.3 (5)	C13—C14—C15—N4	118 (13)
C2—C3—C4—C5	−0.3 (5)	C2—C1—N1—C5	−0.2 (5)
C3—C4—C5—N1	−1.1 (4)	C4—C5—N1—C1	1.3 (4)
C3—C4—C5—C6	178.0 (3)	C6—C5—N1—C1	−177.8 (3)
N1—C5—C6—N2	152.9 (2)	N3—C6—N2—C12	0.7 (3)
C4—C5—C6—N2	−26.3 (4)	C5—C6—N2—C12	−176.9 (2)
N1—C5—C6—N3	−24.3 (4)	C11—C12—N2—C6	179.1 (3)
C4—C5—C6—N3	156.6 (3)	C7—C12—N2—C6	0.2 (3)
C12—C7—C8—C9	−0.3 (4)	N2—C6—N3—C7	−1.3 (3)
N3—C7—C8—C9	178.5 (3)	C5—C6—N3—C7	176.3 (2)
C7—C8—C9—C10	1.1 (4)	N2—C6—N3—C13	172.2 (2)
C8—C9—C10—C11	−0.8 (5)	C5—C6—N3—C13	−10.3 (4)
C9—C10—C11—C12	−0.4 (4)	C8—C7—N3—C6	−177.6 (3)
C10—C11—C12—N2	−177.5 (3)	C12—C7—N3—C6	1.4 (3)
C10—C11—C12—C7	1.3 (4)	C8—C7—N3—C13	8.6 (4)
C8—C7—C12—C11	−0.9 (4)	C12—C7—N3—C13	−172.5 (2)
N3—C7—C12—C11	180.0 (2)	C14—C13—N3—C6	102.0 (3)
C8—C7—C12—N2	178.1 (2)	C14—C13—N3—C7	−85.3 (3)
N3—C7—C12—N2	−1.0 (3)		

### Hydrogen-bond geometry (Å, °)

**Table:**

*D*—H···*A*	*D*—H	H···*A*	*D*···*A*	*D*—H···*A*
N2—H5···O4	0.77 (3)	2.11 (3)	2.885 (4)	179 (4)
C11—H11···O3	0.93	2.54	3.343 (5)	145
C4—H4···O4	0.93	2.62	3.347 (4)	135
C13—H13A···N1	0.97	2.41	2.903 (4)	111
C10—H10···N4^i^	0.93	2.64	3.423 (4)	142
C13—H13B···O2^ii^	0.97	2.60	3.427 (4)	144
C14—H14B···O2^iii^	0.97	2.57	3.483 (4)	157
C2—H2···O1^iv^	0.93	2.62	3.552 (4)	180

Symmetry codes: (i) *x*, *y*+1, *z*−1; (ii) *x*, *y*, *z*+1; (iii) −*x*+1, −*y*+1, −*z*+1; (iv) −*x*, −*y*, −*z*+1.
